# Targeted approaches to delineate neuronal morphology during early development

**DOI:** 10.3389/fncel.2023.1259360

**Published:** 2023-10-03

**Authors:** Bimin Liu, Yuxiao Li, Miao Ren, Xiangning Li

**Affiliations:** ^1^State Key Laboratory of Digital Medical Engineering, School of Biomedical Engineering, Hainan University, Haikou, China; ^2^Key Laboratory of Biomedical Engineering of Hainan Province, School of Biomedical Engineering, Hainan University, Haikou, China; ^3^Research Unit of Multimodal Cross Scale Neural Signal Detection and Imaging, Chinese Academy of Medical Sciences, HUST-Suzhou Institute for Brainsmatics, JITRI, Suzhou, China

**Keywords:** neonatal mice, neural labeling, morphology, neural circuit, development

## Abstract

Understanding the developmental changes that affect neurons is a key step in exploring the assembly and maturation of neural circuits in the brain. For decades, researchers have used a number of labeling techniques to visualize neuronal morphology at different stages of development. However, the efficiency and accuracy of neuronal labeling technologies are limited by the complexity and fragility of neonatal brains. In this review, we illustrate the various labeling techniques utilized for examining the neurogenesis and morphological changes occurring during the early stages of development. We compare the advantages and limitations of each technique from different aspects. Then, we highlight the gaps remaining in our understanding of the structure of neurons in the neonatal mouse brain.

## 1. Introduction

**Figure 1:**
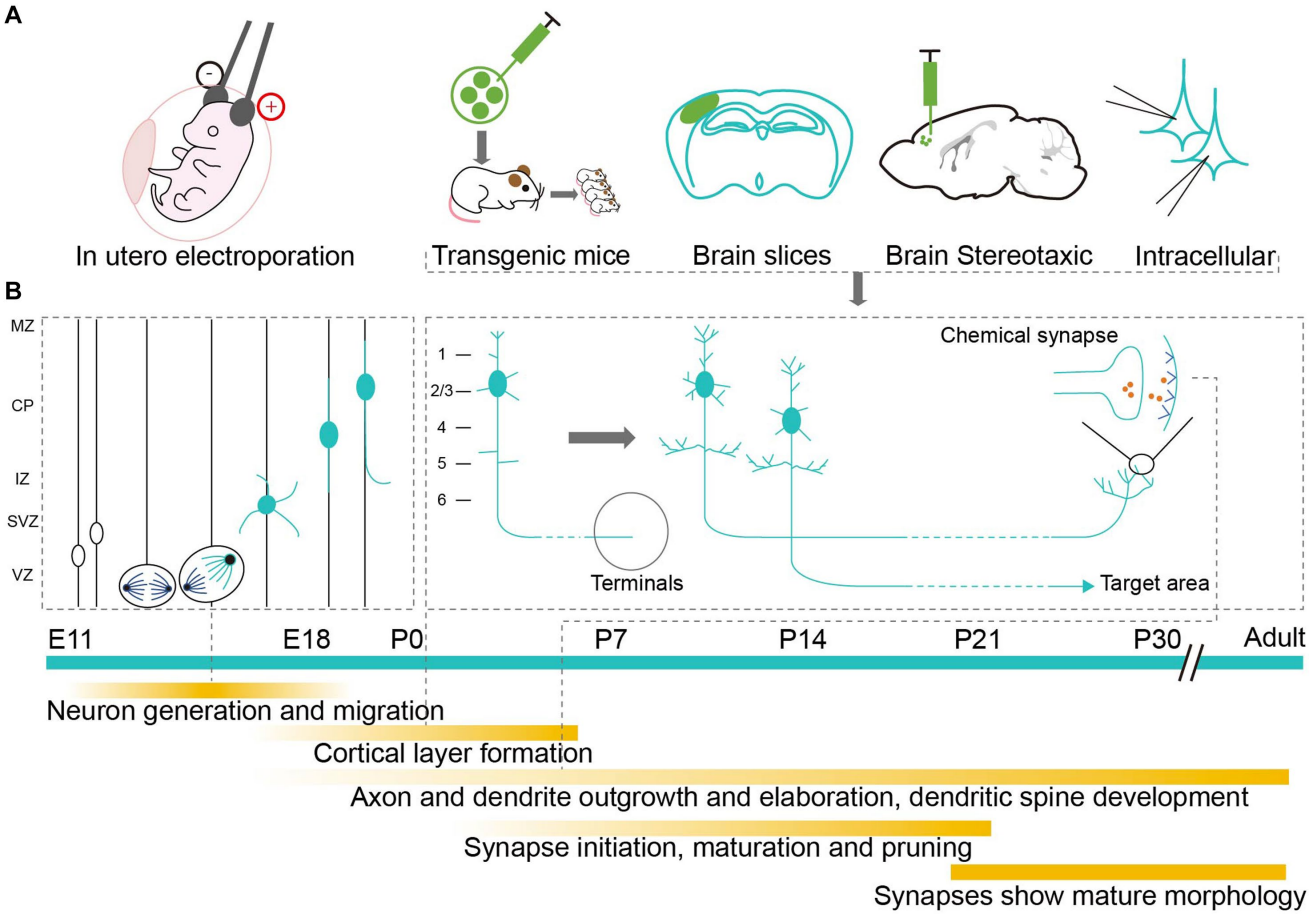
Labeling methods studying the development of neurons in neonatal mouse brain. **(A)** Application of labeling methods at different developmental stages. Markers are delivered to neonatal mouse brain by electrical impulse and injection to label neurons *in vivo* or *in vitro*. **(B)** Milestones of neuron development. The timeline starts from the mid-embryo to the adult. Above the green timeline, it shows the division and migration of neurons during the embryonic period and the growth of pruning of axons, dendrites and synapses during the early postnatal period. Below the green timeline, it shows the detailed development events.

Neurons are fundamental units in the assembly, structure, and function of the brain (Luo, 2021). Realistically visualizing neurons requires researchers to conduct a detailed investigation of each subassembly, with its intricate and exquisite architecture, especially during the complex and rapidly changing development period (Semple et al., 2013; Lim et al., 2018; Cadwell et al., 2019). Utilizing different labeling methodologies can reveal the elaborate developmental mechanisms of neurons (Figure 1A). To be more specific, neurons are derived from the division and differentiation of progenitor cells after migration. Their unique morphological features are usually formed during the postnatal development period (Riccomagno and Kolodkin, 2015; Lim et al., 2018), which involves the growth and pruning of axons, dendrites, and synapses (Inta et al., 2008; Belvindrah et al., 2011; Keil et al., 2017; Kroon et al., 2019; Figure 1B). Labeling technologies can neatly demonstrate any abnormal developmental changes to neurons that may result in abnormal neural connections and lead to long-term or adult behavioral abnormalities (Sizonenko et al., 2005; Rincel et al., 2018). Hence, accurately labeling and analyzing the morphological changes to neurons in the neonatal mouse brain are helpful if we are to further elucidate the cellular mechanisms involved in the development of neural circuits and related diseases (Badea et al., 2003; Luo et al., 2016).

**Figure 2:**
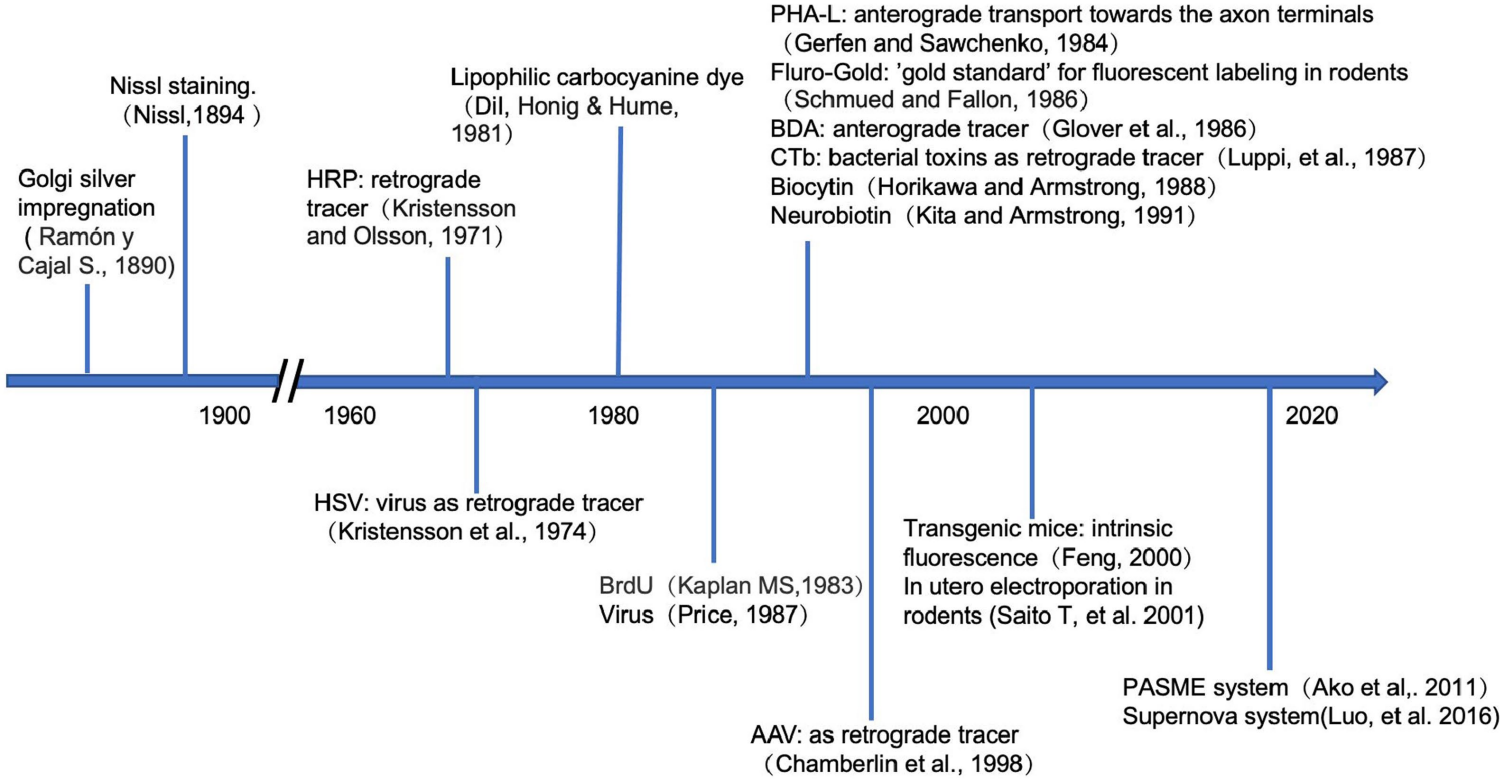
Timeline of the neuronal labeling techniques. Above the horizontal axis, it shows when the traditional tracers were firstly applied in the study of neuroscience. Below the axis, it demonstrates the development of genetic approaches in tracing the neurons.

Labeling methods can be broadly divided into two categories: traditional markers, such as fluorescent dyes, proteins, and bacterial toxins (Ding and Elberger, 2001; Porrero et al., 2010), and genetic labeling. Because of their diverse characteristics, different markers can trace different sections of the neuronal arbors. Genetic labeling can even accurately achieve the visualization of specific types of neurons (Kim et al., 2013; Nakazawa et al., 2018; Olivetti et al., 2020). These labeling techniques provide reliable tools for the precise analysis of neuronal morphologies and developmental history, as is shown in Figure 2.

**Figure 3:**
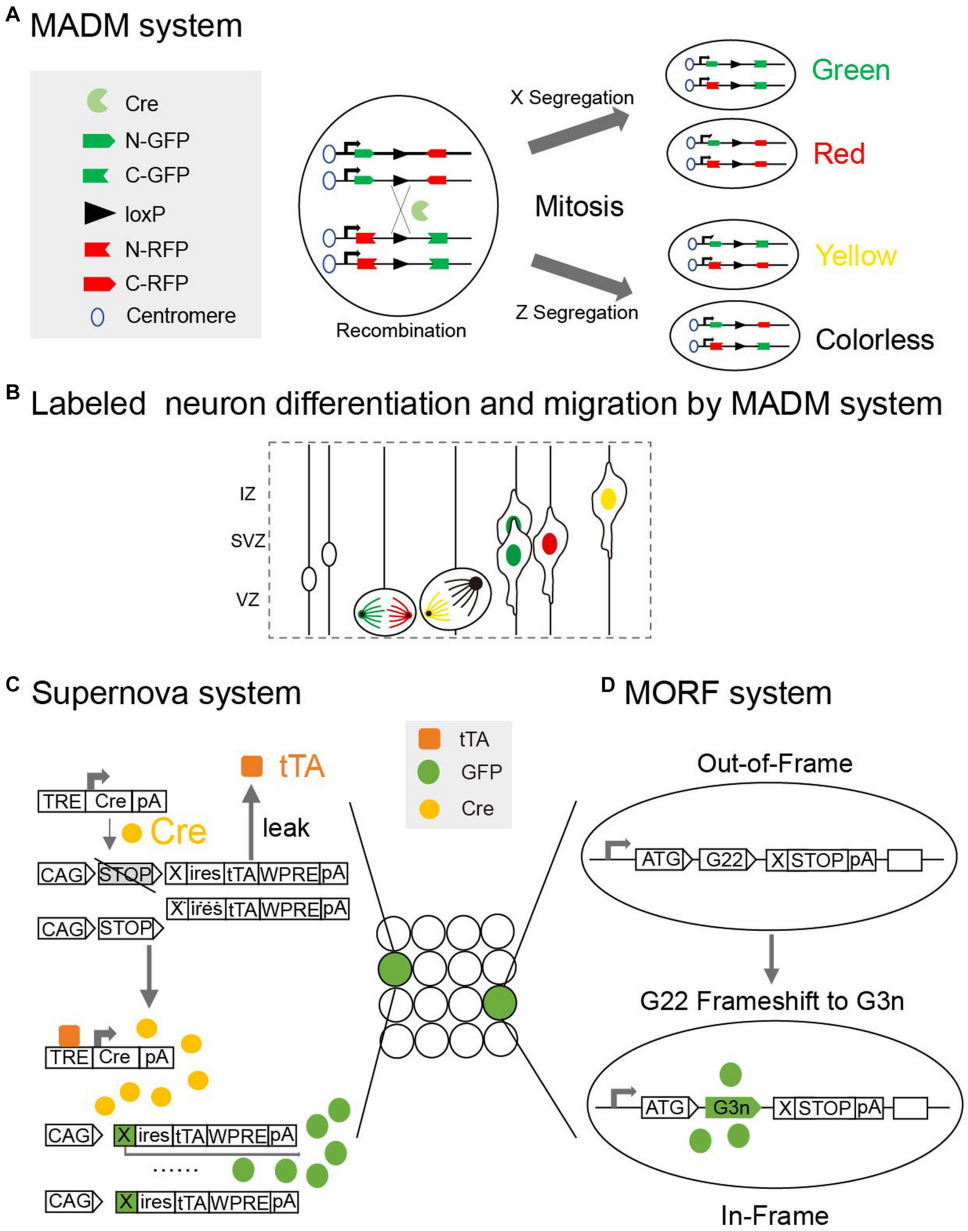
**(A)** Mosaic analysis with double markers (MADM) is a genetic system that allows fluorescent labeling of sparse neurons *in vivo*. Two chimeric marker genes, each containing the N terminus of one marker and the C terminus of the other marker interrupted by a loxP-containing intron, are located on the homologous chromosomes. The expression of green or red fluorescent proteins requires Cre-mediated interchromosomal recombination. X segregation or Z segregation can generate four types of neurons with different markers. **(B)** Diagram showing how MADM system can labeled sister neurons and record their differentiation and migration. **(C)** Supernova system can help to sparse label neurons based on low Tetracycline response element (TRE) leakage. In a small number of neurons with over-threshold leakage, initial tTA (Tetracycline transactivator) -independent weak expression is enhanced by positive feedback along with a site-specific recombination system (Cre/loxP). **(D)** Mosaicism with Repeat Frameshift (MORF) allows a single Bacterial Artificial Chromosome (BAC) transgene to sparse label neurons in mice. A mononucleotide G22 repeat was inserted and only neurons with frameshift of G22 to G3n will express fluorescent signals.

There are still many challenges faced when attempting to label neurons in the newborn mouse brain. Firstly, labeling signals are weaker in the early stages than in adulthood when using genetic tags (Porrero et al., 2010). Secondly, the neonatal mouse brain is fragile to interference. For example, the injection of viral tracers may induce an inflammatory response and microglia activation (Chan et al., 2021), and the electric pulses of *in utero* electroporation (IUE) may lead to embryonic death (dal Maschio et al., 2012). Moreover, different brain nuclei grow at different rates in the neonatal brain, making it difficult to precisely localize neurons (Paxinos et al., 2020). To address these issues, researchers have optimized multiple aspects of the strategies by, for example, improving the efficiency and stability of markers, upgrading the loading methods and adopting genetic systems like Mosaic analysis with double markers (Figures 3A,B). These innovations have greatly advanced our knowledge of developmental neurons in the neonatal mouse brain.

In summary, labeling neurons in the neonatal mouse brain is key to studying the development of neural circuits. Accurately marking the morphology of specific neurons in the neonatal brain is conducive to answering the questions of how and when neurons connect to perform specific functions. It also helps to refine our understanding of the mechanisms by which abnormalities in neural circuits are caused by environmental or gene mutations during the sensitive neonatal period and to test the effects of early treatments and interventions on cognitive disorders and mental diseases. In this review, we summarize the techniques used for labeling neural morphology at different developmental stages. We also analyze the advantages and limitations of the different labeling techniques, providing directions that can be taken by researchers to further explore the mechanisms of neural development.

## 2. Chemical tracers

### 2.1. Labeling neurons of young mice *in vitro*

At the end of the nineteenth century, the classic Golgi stain, which is still widely used to study the development of neurons, was formulated. The principle of sparse labeling is that the dark brown precipitation generated by the reaction between the potassium bichromate solution and silver ions randomly labels a relatively small number of neurons, particularly the dendrites. Using Golgi staining, researchers have recorded the morphological changes to dendrites occurring in the early developmental stages (Ivy and Killackey, 1981; Niwa et al., 2010). The use of hematoxylin and eosin (HE) stains the cell nuclei within brain tissue blue with hematoxylin, and proteins are stained pink with eosin (Fischer et al., 2008; Picut et al., 2021). HE staining can detect morphological changes to neurons in model mice during their early developmental stages (Zhou et al., 2018). The Nissl staining method uses basic dyes like cresyl violet acetate or toluidine blue to permanently stain nucleic acids within neurons in the brain (Paul et al., 2008; Smiley et al., 2023) and is used for detecting the morphology and pathology of neuronal tissues and understanding the cytoarchitecture of different brain areas (Kawase-Koga et al., 2009). Immunohistochemical staining uses antibodies to target specific antigens in order to show morphological changes to neurons at different developmental stages (Lee et al., 1998). Nevertheless, these classic staining methods are laborious. They are limited in use to fixed brain samples, instead of live ones.

### 2.2. Labeling neurons of young mice *in vivo*

In the second half of the twentieth century, many traditional neural tracers were developed that can label neurons *in vivo*. Horseradish peroxidase (HRP) is one of the earliest markers used in the morphological study of neuronal axons and is still being used in labeling neonatal axons (van der Togt and Feirabend, 1990; Sohur et al., 2014; Harb et al., 2016; De León Reyes et al., 2019; Saleeba et al., 2019). Phytohemagglutinin (Phal) (Fish et al., 1991) is a long-lasting tracer employed for labeling neonatal axons. Biocytin and neurobiotin can also clearly label the dendritic and axonal structure of neurons (Staiger et al., 2004; Moreno-Velasquez et al., 2020). Biotinylated dextran amine (BDA) can be used to detect the normal morphological development of axons and dendrites in neonates (Ding and Elberger, 2001; Sugar and Witter, 2016; Kroon et al., 2019) and abnormal morphology in disease models (van Velthoven et al., 2012). Fluorescent dyes, such as Fluro Gold (Hartung et al., 2016) and Fast Blue (Lopez-Roman et al., 1993), are absorbed by axon terminals and accumulate in the soma through retrograde transport, thus they highlight the dendrites of neurons involved in specific loop connections. Unfortunately, high doses of dyes like Fluro Gold may lead to the death of young mice (Hu H. et al., 2021).

Injecting chemical markers into the ventricle can label massive progenitor cells, with some of the markers being passed onto the progeny. Thymidine analogs like bromodeoxyuridine (BrdU) are commonly used markers (Miller and Nowakowski, 1988) that can be inserted into the double-strand DNA of progenitors during S-phase (Inta et al., 2008). However, they label neurons that have already been in the migration state, which means that intact migration and differentiation pathways cannot be fully traced. To overcome this problem, researchers developed Flash-tag (FT) technology. After injection into the ventricles, carboxyl fluorescein (CFSE) can bind to the proteins in M phase progenitors and their progeny. The specificity of CFSE for M-phase progenitors is the result of the soma of these cells being transiently exposed to the CFSE injected into the cerebrospinal fluid (Telley et al., 2016; Govindan et al., 2018; Yoshinaga et al., 2021). These chemical markers combine with specific molecules in progenitor neurons but are diluted as the cells undergo rapid division.

### 2.3. Labeling specific neurons combined with physical methods

Single-cell injection of traditional chemical tracers can achieve the goal of targeting specific individual neurons in neonatal mouse brains (Larsen and Callaway, 2006). Anterograde tracers such as biocytin (Moreno-Velasquez et al., 2020), biotinylated glucosamine (Cunningham et al., 2002; Staiger et al., 2004), and carbocyanine dyes (red fluorescent Dil and green fluorescent Dio) (Lee et al., 2005; Lickiss et al., 2012; Sohur et al., 2014) can mark the axons and dendritic structures of developing neurons. By utilizing physical methods like single-neuron injection, researchers can locate specific neurons within a targeted area. As it is challenging to perform single-neuron injection, more labeling methods targeting specific neurons are needed.

Chemical tracers are diverse, highly sensitive, and can be transported retrograde or anterograde by being attached to different vectors at different rates (Table 1). However, some types of traditional chemical tracers are toxic to neurons, prone to dye leakage and the mislabeling of passing fibers, and fail to identify neurons that express specific molecular markers. These limitations may reduce their reliability and accuracy for labeling neurons in neonatal mice.

**Table 1:** Parameters of the neural tracers: “−” indicate no effect, “+” “++” “+++” show the strength of labeling effect.

	Works in				Quality of labeling				Uptake		References
Tracer	Vivo	Fixed tissue	Live slices	Dissociated cells	Soma	Dendrites	Axon	Transport	Fiber of passage	Stability	
FB	++	−	−	−	+++	+	−	R	+	++	Lopez-Roman et al. (1993)
FG	+	−	−	−	+++	++	−	R	+	++	Hartung et al. (2016)
Fluro-Jade B	++	−	−	−	++	++	+	R	+	+	Sizonenko et al. (2005) and Picut et al. (2021)
HRP	+++	+/−	−	−	+	+	++	A	++	++	Ivy and Killackey (1981), van der Togt and Feirabend (1990), Sohur et al. (2014), Harb et al. (2016), De León Reyes et al. (2019), and Saleeba et al. (2019)
CTB	+++	−	−	−	+++	+++	+++	R	+	+++	Heise and Mitrofanis (2004)
BDA	+++	+	+	+/−	+++	++	+++	A/R	+/−	+++	Ding and Elberger (2001) and van Velthoven et al. (2012)
Biocytin	+++	+	+	−	+++	++	+++	A/R	+/−	+++	Cunningham et al. (2002) and Staiger et al. (2004)
Neurobiotin	+++	?	++	−	+++	++	+++	A/R	+/−	+++	Kohara and Okada (2023)
DiI	++	+++	+++	++	+	+	++	A	+++	++	Lee et al. (2005), Lickiss et al. (2012), and Sohur et al. (2014)
DiO	++	++	++	++	+	+	++	A	+++	++	Sohur et al. (2014)
PHA-L	++	+++	++	+	+	+	+++	A	+/−	+++	Fish et al. (1991)

“R” stands for retrograde while “A” stands for anterograde.

## 3. Genetic engineering technology

### 3.1. Viral tracers

Neurotropic viruses are widely used vectors for labeling neurons in young mice. As the first viral tracers used to label the soma of the neonatal mouse, retroviruses can permanently integrate the genome they carry into host neurons after mitosis, achieving a long-term and stable fluorescent signal (Price et al., 1987; Badea et al., 2003; Yu et al., 2009). Despite this, the infection efficiency of retroviruses is relatively low. In addition, gene insertion by retroviruses is random, which may lead to the mutation of endogenous genes, resulting in unexpected abnormal changes to neurons.

Adeno-associated viruses (AAV) have the advantages of versatility and multiple serotypes, rendering them a popular tool in the field of neuroanatomy (Castle et al., 2016; Hammond et al., 2017). A viral tracer can be injected into neonatal mouse brains, facilitating the visualization of neurons in about 2 weeks (Cheetham et al., 2015; Chen et al., 2018). Through the implementation of an exceptional extrinsic transgenic system and the utilization of various viral tracers, one can investigate specific morphological transformations in distinct neuron types, such as pyramidal neurons and interneurons, in the postnatal period (Kim et al., 2013). Furthermore, AAV can carry a supernova system (Luo et al., 2016) that makes use of the low leakage characteristics of the tetracycline response element for select marking (Figure 3C). The clustered regularly interspersed short palindromic repeats (CRISPR)-CRISPR associated (Cas)9 system can be used to integrate specific genes and viral tracers into the genomes of target neurons with unprecedented speed and precision, allowing researchers to visualize developing neurons by identifying the specific proteins they express (Uemura et al., 2016). In addition, diluted viruses such as AAV (Gibson and Ma, 2011; Kim et al., 2013), Sindbis virus (Lendvai et al., 2000), and retroviruses (Kerloch et al., 2019) may be used to sparsely label neurons in neonatal mice.

Viral tracers are a popular tool in the field of neurodevelopment. Nevertheless, there are still several limitations associated with the application of viral tracers in neonatal mouse brains. Only a few studies have revealed details of the morphological development of neurons during the early postnatal period using viral tracers (Pilpel et al., 2009), as it generally takes about 2 weeks for viral tracers to express detectable signals in mouse brains. There is some debate as to whether the use of viral tracers causes the inflammation and gene-coding-related abnormal growth of neurons (Uyaniker et al., 2019). In addition, the standard procedure used for the stereotactic injection of viruses into adult mice may not be suitable for use on neonatal mice. It is more complicated to anesthetize, immobilize, locate brain regions, and precisely inject viruses into neonatal mice (Ho et al., 2020; Olivetti et al., 2020).

### 3.2. Transgenic mice

Transgenic mice provide an option for non-invasive labeling. A wide variety of transgenic mice can be generated by prokaryotic injection, bacterial artificial chromosomes (BACs), or knock-in techniques. In the study of neuron lineage tracing, transgenic mice have been generated to target specific types of neurons that develop after embryonic development (Gao et al., 2014; Huang, 2014; He et al., 2016).

In the case of transgenic mice, different insertion sites and copies of foreign genes can be used to enable the sparse and random labeling of neurons. Driven by specific promoters, genes in these transgenic mice express site-specific recombinases that identify specific DNA sequences and activate the expression of fluorescent proteins (Huang et al., 2002). For instance, the dendrites of neurons in the cortex and hippocampus have been visualized in transgenic mice that express fluorescent proteins regulated by the Thy1-promotor (Feng et al., 2000). Mosaic analysis with double markers (MADM) is a genetic system that allows for the fluorescent labeling of neurons *in vivo* and has been used in the tracing of neuron lineages (Espinosa and Luo, 2008; Figure 3A). In MADM, two chimeric marker genes interrupted by a *loxP*-containing intron are located on homologous chromosomes, and the expression of the fluorescent proteins requires cyclization recombinase (Cre)-mediated interchromosomal recombination. Transgenic mice used for lineage tracing with the MADM (Figure 3B) system can also be used to visualize partial neuronal dendrites in neonatal mice brains (Yu et al., 2009; Tasic et al., 2012). Cyclization recombinase estrogen receptor (Cre-ER) transgenic mice, which express fusion proteins containing estrogen receptors and Cyclization recombinase (Cre), have neurons that are sparsely labeled under the induction of tamoxifen (Badea et al., 2003). The Flp/FRT recombinase system is based on the same strategy as the Cre/loxp system, but it has only been applied in a few cases for visualizing the neuronal morphology in neonatal mice brains (Nern et al., 2011). Mosaicism with repeat frameshift (MORF) allows a single Bacterial artificial chromosome (BAC) transgene to sparsely label neurons in mice. A mononucleotide G22 repeat is inserted, and only neurons with the G22 to G3n frameshift express the fluorescent signals. With the aid of *in vivo* imaging techniques, transgenic mice facilitate the dynamic long-term tracking of development of dendrites (Portera-Cailliau et al., 2005; Zuo et al., 2005).

Despite the advantages, labeling signals in transgenic mice maybe unstable in the early developmental stages. Transgenic mice like mGFP, L21, may express relatively weak signals during the early postnatal period (Porrero et al., 2010). The temporal expression of these signals may be caused by the gradual promotion of the expression of genes, such as the Thy-1 promotor. Or it could be attributed to the insertion of foreign genes and the timing of the expression of specific markers in various neuron types. For example, only the dendrites and soma-proximal axons of dopamine neurons can be observed in the early postnatal period of Mosaicism with repeat frameshift (MORF) transgenic mice (Lu and Yang, 2017). BAC transgenic mice, which are based on homologous recombination, show weak signals for labeled GABAergic neurons (GABA: Gamma-aminobutyric acid) during the early postnatal period (Chattopadhyaya et al., 2004). CreER transgenic mice, which need to be treated with tamoxifen before they activate the expression of specific genes, do not display strong signals in their cholinergic neurons until 2 or 3 weeks after birth (Rotolo et al., 2008). While the combination of transgenesis and viral tracers increases the flexibility of sparse labeling (Le Magueresse et al., 2011; Zhang et al., 2017), the problem of the delayed expression of genetic tags remains unresolved. Further improvements are required to optimize the labeling efficiency of transgenic tools.

### 3.3. *In utero* electroporation

*In utero* electroporation (IUE) is a popular labeling method that has been in development since 2001 (Saito and Nakatsuji, 2001). Gene vectors can be injected into the ventricle as early as the embryonic stage. Then, electrical pulses induced by the electrodes on both sides of the embryo’s head help transduce the vectors into the progenitor neuron population within a specific brain region, such as the cortex (Niwa et al., 2010) or hippocampus (Navarro-Quiroga et al., 2007). As the progenitor neurons continue to divide, foreign genes are passed to daughter neurons, which differentiate and migrate to targeted regions. For mice, the optimal surgical period for IUE is between 10.5 and 16.5 embryonic days (Yoshida et al., 2010). In this regard, IUE can alleviate the issue of delayed gene expression in the postnatal period. The vectors carrying fluorescent protein genes can be transfected into specific neurons during the embryonic period to enable the tracking of axon fiber bundles during development (Wang et al., 2007; Chen et al., 2008; Ka and Kim, 2016; Kerloch et al., 2019). However, the transfection range of IUE is limited, and electrical impulses may affect the normal development of neurons (Azzarelli et al., 2017).

A notable advantage of transgenic labeling technology lies in its ability to precisely detect molecular markers in distinct types of neurons. This allows for controlled detection signals to be expressed solely in targeted neuron subtypes. IUE provides a reliable protocol for delivering genetic tags to specific types of neurons. By selectively targeting neural progenitors across distinct developmental stages, IUE can facilitate the investigation of unique groups of newborn neurons that will subsequently migrate to various brain regions (Mizuno et al., 2010). Minimal amounts of plasmid (Pacary et al., 2012; Sugiyama et al., 2020) are needed, and single-neuron electroporation can be used to label a relatively small number of neurons (Uesaka et al., 2005). Vectors carrying the supernova system can be delivered to specific neurons for morphological labeling (Mizuno et al., 2018). Moreover, using CRISPR/Cas9 combined with piggyBac transposase technology, researchers can track the development of specific neocortical progenitors with IUE (Chen et al., 2015). The promoter-assisted spares-neuron multiple-gene labeling using *in utero* electroporation (PASME) system (Ako et al., 2011) utilizes the Thy1S promoter, the Cre-loxP system, and IUE. The Thy1S promoter is highly selectively expressed in the postnatal neocortex and can induce GFP expression in only a limited number of neurons (Figure 3D).

It is vital that we discover what links the different types of morphological changes that occur in the basic unit of signal transmission in the brain, the neuron, with the connections and functions of complex neural circuits. The development of genetic engineering technology provides abundant options for scientists to track changes to the morphology of neurons during the developmental period (Table 2). Genetic labeling methods can be used to target different types of neurons, but it is difficult to target single neurons by physical methods, and there is a lack of stable signal during the early developing period. Consequently, to obtain a continuous and comprehensive map of the morphological development of various neuron types, further research is required.

**Table 2:** Summary of viral, plasmid, and genetic labeling methods: advantages and warning.

Method	Means of delivery	Cell types targeted	Advantages	Caveats	References
Retro-virus	Stereotaxic, focal injection	Dividing cells	~7.5 kb insert	Genomic integration disrupts host DNA at insertion site	Price et al. (1987), Suzuki and Goldman (2003), Yu et al. (2009), Artegiani and Calegari (2013), Zhang et al. (2017), Kerloch et al. (2019), and Program et al. (2022)
			Persistent genetic alteration of dividing transduced cells	Focal injection by its nature causes lesion	
Adeno associated virus	Stereotaxic, focal injection	Broad range	~8 kb insert	Take more time for expression	Gibson and Ma (2011), Cheetham et al. (2015), Luo et al. (2016), Chen et al. (2018), Ren et al. (2018), and Hu S. et al. (2021)
			Higher efficiency of infection	Focal injection by its nature causes lesion	
Plasmid	Stereotaxic, focal injection, electroporation	Broad range	Permits larger inserts	Cannot replicate autonomously	Uesaka et al. (2005), Yoshida et al. (2010), Belvindrah et al. (2011), Lickiss et al. (2012), and Szczurkowska et al. (2016)
Trans-genic mice	–	Broad range	Intrinsic fluorescence	Higher breeding cost	Feng et al. (2000), Chattopadhyaya et al. (2004), Madisen et al. (2010), Porrero et al. (2010), Le Magueresse et al. (2011), van Velthoven et al. (2012), and Lu and Yang (2017)
			Strong compatibility (combined with virus and plasmid labeling)		

## 4. Discussion

The development of neurons is a critical step in the establishment of precise neural circuits. Over the past several decades, a variety of labeling techniques have been invented that allow us to visualize the morphology of neurons as they develop. Thus, researchers have found that different neurons form distinct structures and exhibit different dynamics. However, current labeling techniques do not yet satisfy the need to track all types of neurons from their formation in the embryonic stage to maturation in adulthood.

Adjustments to surgical protocols and the optimization of equipment continue to improve the utility of labeling technology. For IUE, triple-electrode probes have been developed to greatly extend the range of suitable transfection areas (dal Maschio et al., 2012; Szczurkowska et al., 2016). Dual IUE is used to label different neurons in a spatially and temporally specific manner (Zhang et al., 2021). In addition, the simultaneous application of multiple labeling methods, such as a combination of transgenic mice and viral tracers, helps to separately label specific subtypes of neurons with different spatial and temporal characteristics (Madisen et al., 2010). The optimization of workflow can be useful in enriching the labeling options for neurons in neonates while improving the labeling efficiency of the markers.

One major main technical barrier to overcome in this field is our ability to balance the long-term stable expression of markers with the sparse labeling of specific neurons during different development stages. Usually, the insertion and expression of foreign genes takes a certain amount of time, which may result in us missing the short time window for the optimal observation of developing neurons. For example, AAV does not express strong enough signals until a few weeks after injection. To accelerate the expression of labeling signals, especially during the early postnatal period, transgenic systems can be updated by increasing the number of gene copies encoding fluorescent proteins or developing more efficient sparse labeling constructs. The Brainbow system can be employed to label individual neurons with distinguishable colors through the stochastic expression of several fluorescent reporter transgenes, and neurons expressing a particular color thus share a common lineage (Livet et al., 2007). With further advances in these imaging technologies, these systems may continue to be attractive choices for tracing several types of neurons simultaneously.

The development of new labeling technology is always accompanied by advances in imaging technology. Initially, neural tracers were limited in use to fixed tissues or thin slices *in vitro* until dyes with higher biocompatibility and *in vivo* imaging techniques were invented. Nowadays, technologies such as intracranial multiphoton microscopy and head-mounted microscopes offer us the ability to observe the development of living animals in real-time. Secondly, the retention of precise fluorescence signals in neurons within intact brains provides more possibilities for scientists to conduct the anatomical dissection of neural systems and even study the fine morphology of individual neurons (Ren et al., 2018, 2021). Fluorescence micro-optical sectioning tomography (fMOST) enables researchers to benefit from three-dimensional imaging with single-cell resolution at the mesoscopic scale (Li et al., 2010; Gong et al., 2016; Zhong et al., 2021). This greatly facilitates the visualization of the complete morphology of single neurons and even synaptic projection patterns and lesion characteristics in the whole brain (Oh et al., 2014; Li et al., 2018; Sun et al., 2019, 2022; Tian et al., 2022). Advanced imaging techniques such as these permit researchers to better exploit labeling techniques when exploring the developmental mechanisms of neurons.

With the ongoing development of labeling and imaging technologies, researchers are beginning to integrate databases on neuron morphology, genome, proteins, etc., to compile a more detailed map of the developing brain. The results have already revealed the abnormal changes to neonatal neurons in mice with pathologies such as autism (Varghese et al., 2017). These abnormal neuronal morphological changes are closely related to impairments to the cognitive functions shown by pathological mice (Druart et al., 2021). The scientific community now has a deeper understanding of the mechanisms involved in brain development, which provides a solid theoretical foundation upon which cures for neurodevelopmental disorders and neurodegenerative diseases can be based.

## Author contributions

BL: Writing – original draft. YL: Writing – review & editing. MR: Writing – review & editing. XL: Writing – review & editing.

## Funding

The author(s) declare financial support was received for the research, authorship, and/or publication of this article. This work was financially supported by STI2030-Major Projects (no. 2022ZD0205201), National Nature Science Foundation of China (no. T2122015), CAMS Innovation Fund for Medical Sciences (no. 2019-I2M-5-014), and the Hainan Provincial Natural Science Foundation of China for High-level Talents (no. 821RC531).

## Conflict of interest

The authors declare that the research was conducted in the absence of any commercial or financial relationships that could be construed as a potential conflict of interest.

## Publisher’s note

All claims expressed in this article are solely those of the authors and do not necessarily represent those of their affiliated organizations, or those of the publisher, the editors and the reviewers. Any product that may be evaluated in this article, or claim that may be made by its manufacturer, is not guaranteed or endorsed by the publisher.
